# Effectiveness of STEM based workshop for deaf education: Exploratory study

**DOI:** 10.1016/j.heliyon.2024.e36012

**Published:** 2024-08-10

**Authors:** Ruba Anwar, Abubaker M. Elbashir, Rana Magdy, Zubair Ahmad, Noora J. Al-Thani

**Affiliations:** Qatar University Young Scientists Center (QUYSC), Qatar University, 2713, Doha, Qatar

**Keywords:** STEM workshop, Deaf education, Creative problem solving, Effectiveness, STEM skills

## Abstract

STEM education for deaf students aims to engage and include intellectual and experiential learning other than normal classrooms. These programs improve students' critical thinking, problem-solving, creativity, and complex decision-making, which are essential for academic and life success. This study aims to explore several aspects of a STEM based workshop including problem solving skills, STEM skills, subject knowledge, and effectiveness of the workshop for a group of 27 deaf students. The workshop spanned five consecutive days and focused on problem-solving principles within the context of global warming. Moreover, in this study, the Creative Problem-solving approach developed by Osborn and Parnes was implemented to measure improvement of the constructs above, through a post questionnaire. Cronbach's alpha and McDonald's Omega coefficient exceeded .7 for each construct. The data obtained from the questionnaire demonstrated a random distribution of data according to the Shapiro-Wilk test performed (p < 0.05), leading to the use of non-parametric analysis tools. The results based on the non-parametric test analysis (Kruskal Wallis test) show that high school students' problem-solving abilities improved despite the data's randomness (Mean rank = 16.72). The workshop was enhanced for the Preparatory students, who tended to gain more STEM skills and problem-solving abilities from it (Mean rank = 14.75). It also improved the knowledge and STEM skills of Primary-stage students (Mean rank = 18.13 and 18.06, respectively). This study contributes to the existing body of literature by examining how addressing challenges of global warming can enhance various abilities among deaf students.

## 1 Introduction

In the contemporary era, the field of deaf education acknowledges the significance of imparting students with a diverse array of abilities and proficiencies commonly denoted as "21st-century skills". Providing education to deaf students is of paramount importance in promoting the development of advanced cognitive abilities among these students. Higher-order skills extend beyond fundamental knowledge and encompass critical thinking, creativity, problem solving, and advanced cognitive capabilities. The aforementioned skills extend beyond conventional academic knowledge and prioritize equipping students for achievement in a progressively intricate and interrelated global context [1]. It is important to note that the development of higher-order skills varies between individuals, and that support and education can help to improve these skills. Deafened students benefit from educational programs that emphasize the development of critical thinking, problem solving, creativity, and other higher-order skills. Additionally, early intervention and ongoing support can help address any skill deficits and promote overall success and well-being for deafened individuals. In this context, problem-solving skills are important and affect children as they provide the foundation for academic success, social interactions, and life skills development. Morris et al. [2] suggested successful STEM cooking program learning supports. They reviewed data indicating authentic, social, and collaborative learning activities that use culturally relevant techniques and knowledge work best. They suggested that cooking affords authentic, culturally relevant learning opportunities and natural learning and engagement supports. They found that cooking might assist STEM learning to overcome scalability issues and enhance interest and long-term participation. In their work with K-12 educators to increase their understanding and encourage activities that purposefully influence kids' STEM interests and career trajectory, Amari et al. [3]discussed the obstacles of introducing STEM experiences. Initial classification and analysis of document materials and transcripts revealed school educators' viewpoints on STEM learning. They show how distinctive school factors (such as support from numerous educators, defined STEM club leadership responsibilities, and targeted recruitment techniques) affect STEM learning opportunities. They demonstrate how action research and a community of practice might improve school-based, STEM programs. An STEM learning strategy by Cohen et al. [4]was used in the context of deaf education, specifically targeting a group of deaf kids aged 16–20 years old. This approach was executed through a 5-day science camp, during which the participants actively participated in various STEM activities during the duration of the program. The study highlighted the increasing theme of deaf students within inclusive and accessible STEM learning environments, focusing on three key aspects: language representation, cultural role models, and self-recognition. The results additionally underscore the significance of developing an inclusive educational environment that promotes equity for underprivileged students within the framework of science education.

## 2 Literature review

### 2.1 STEM approach in deaf education

Science, Technology, Engineering, and Mathematics (STEM) education has gained significant attention in recent years due to its importance in preparing students for the demands of the 21st-century workforce [5]. However, deaf students often face unique challenges in accessing STEM education [6]. This literature review aims to explore the current state of STEM education for deaf students, the challenges they face, and the strategies used to overcome these challenges. Deaf students encounter several barriers when it comes to STEM education. One of the primary challenges is the lack of accessible content and resources [7]. Many STEM materials, such as textbooks and videos, are not designed with deaf students in mind and may lack proper captioning or visual aids [8,9]. Additionally, deaf students may struggle with the technical vocabulary used in STEM subjects, as they may not have had sufficient exposure to these terms in their early education [10,11]. Another challenge is the limited number of qualified STEM teachers who are proficient in sign language or have experience working with deaf students [12]. This can lead to communication barriers and a lack of understanding between teachers and students [13].

There are different strategies that have been proposed for delivery of STEM approach for deaf students. One approach is to use visual aids and manipulatives to make STEM concepts more accessible to deaf students [7]. For example, using 3D models, diagrams, and interactive simulations can help students had better understand complex ideas [14,15]. Another strategy is to provide professional development opportunities for STEM teachers to learn about deaf culture, sign language, and effective teaching strategies for deaf students [16,17]. This can help bridge the communication gap between teachers and students and create a more inclusive learning environment [18]. Collaborative learning and peer support have also been found to be effective in promoting STEMeducation for deaf students. Encouraging group work and providing opportunities for deaf students to work alongside their hearing peers can foster a sense of inclusion and improve academic performance [10,19]. The use of technology has also proven to be a valuable tool in STEM education for deaf students. Assistive technologies, such as real-time captioning, sign language videos, and virtual reality simulations, can enhance the learning experience and make STEM content more accessible [7,20].

Environmental education is essential for cultivating a sense of accountability and guardianship towards the natural environment among all students, including those with hearing impairments. By integrating a STEM (Science, Technology, Engineering, and Mathematics) methodology, environmental educational programs can offer deaf students practical, hands-on learning experiences that foster critical thinking, problem-solving skills, and environmental knowledge. The benefits, challenges, and best practices of integrating environmental education programs with a STEM focus for deaf students are explored in this study. STEM-based environmental educational programs offer a promising approach for engaging deaf students in meaningful, accessible, and empowering learning experiences that foster environmental literacy, critical thinking, and problem-solving skills [21]. These programs incorporate hands-on activities, technology integration, collaborative projects, and inclusive practices to create accessible and culturally responsive learning environments [22,23]. The success of these programs relies on the commitment and support of educators, administrators, and community partners, as well as professional development and training in evidence-based practices for deaf education [24]. As research continues to evolve, prioritizing the unique needs and strengths of deaf students in the design and implementation of STEM-based environmental education programs is essential [25].

### 2.2 Skill development for deaf students

STEM workshops can be a powerful tool for enhancing both subject knowledge and problem-solving skills for deaf students. They frequently encounter distinctive obstacles when it comes to accessing STEM education and cultivating these abilities. These challenges arise from communication barriers, a scarcity of accessible resources, and a lack of opportunities for inclusion [26]. STEM workshops have become a valuable resource for improving subject knowledge, fostering problem-solving skills, 4C skills [27], and involving deaf students in practical learning experiences. Interactive STEM workshops and hands-on learning can help deaf students learn and solve problems. A (laboratory science technology) program that engaged deaf students in hands-on experiments and projects improved STEM understanding and problem-solving, according to Pagano et al. [7]. Marschark et al. [10] stress the importance of giving deaf students STEM opportunities to solve real-world problems. Deaf students can learn STEM subjects and develop practical skills by participating in interactive activities and authentic problem-solving scenarios. Workshops must provide accessible content and use communication strategies tailored to deaf students to improve STEM subject knowledge and problem-solving skills. Knoors and Marschark [28] recommend using diagrams, charts, and 3D models to help deaf students understand STEM concepts and problem-solving. For clear communication, workshop facilitators should speak sign language or use interpreters. Deaf workshop participants can also benefit from video captions, real-time captioning, and written materials [9]. Deaf students can learn and solve problems in STEM workshops that encourage collaboration and peer interaction. Coding and robotics can improve students' digital literacy and STEM learning and problem-solving [14]. STEM workshops led by successful deaf STEM professionals help deaf students see themselves as capable of pursuing STEM careers and motivate them to engage with STEM subjects and solve complex problems [29]. STEM workshops also foster a sense of belonging and engagement, which can lead to long-term success.

### 2.3 STEM workshop effectiveness

It is crucial to evaluate the efficacy of STEM workshops for deaf students as they grow in popularity as a way to improve their subject knowledge and problem-solving abilities. Evaluating the impact of these workshops is crucial for understanding their benefits, identifying areas for improvement, and justifying the allocation of resources. Measuring the effectiveness of STEM workshops for deaf students requires a multifaceted approach that includes appropriate assessment strategies, evaluation of learning outcomes, affective factors, long-term impact, and continuous improvement. Pagano et al. and Fobi et al. [30,31] emphasize the importance of using multiple, accessible, and culturally responsive assessment methods, such as pre- and post-workshop surveys, student portfolios, and performance-based assessments.

The development of participants' skills in the STEM (Science, Technology, Engineering, and Mathematics) fields has been shown to benefit greatly from participation in informal STEM workshops, which have been shown to be highly effective in this regard by a number of different research. According to the findings of research carried out by Karan and Brown [32], these types of workshops offer participants a structured and interactive environment in which they can participate in real-world problem solving scenarios, which in turn helps to foster creativity. In addition, Sahin [33] found that these workshops foster teamwork and essential STEM skills, which are both important for the future of STEM. Participants will develop specific problem solving techniques and the confidence to tackle complex STEM challenges because of their participation. Additionally, the participation in hands-on activities and practical exercises during these workshops is found to significantly contribute to the acquisition and retention of STEM skills, as highlighted by Raviv [34]. Informal STEM workshops provide an invaluable educational platform for the enhancement of problem solving skills and STEM skills, thereby providing a foundation for success in careers and academic pursuits related to STEM. Collaborative problem solving which is an approach used in STEM workshops has been implemented using meta-analysis of 36 pieces of the literature revealed in worldwide educational periodicals during the 21st century to identify the effectiveness [35]. Their results concluded that the collaborative problem solving could enhance the critical thinking and showed how it was developed. González et al. [36] conducted a study to assess the effectiveness of online problem solving STEM based workshop in enhancing academic performance. The objective of their study was to determine the magnitude of the impact of homework practice using exercises generated by the ‘e-status' platform on students enrolled in five Engineering programs. The subject topics were divided into two distinct blocks, with each block consisting of nine probability problems. The results of their study offer empirical support for the notion that engaging in a limited amount of online activities can have a beneficial effect on students' academic performance.

## 3 Aim of the study

This research gives a complete picture of how an STEM based workshop for students with hearing disabilities was done, how it was performed, and how well it functioned. The workshop provided hands-on activities and experiments based on the STEM approach that were meant to teach students about global warming and challenge them to come up with innovative solutions to solve problems. This study utilizes quantitative analyses to contribute a novel perspective on the integration of STEM based learning, into four distinct constructs: problem-solving skills, problem solving for STEM skills development, subject knowledge, and general effectiveness of workshop. The objective is to enhance the existing teaching methods employed in special education for students with disabilities, with a particular focus on those with hearing impairments, in order to optimize their learning experience.

The research question (RQ) that the study addresses is:

“Does the STEM based workshop have an impact on deaf students' problem-solving skills, STEM skills, and subject knowledge and does this reflect in the effectiveness of the workshop?”

## 4 Theoretical and conceptual framework

The theoretical foundation of the study is supported by the creative problem solving (CPS) theory. The Creative Problem Solving (CPS) model is a systematic approach that promotes diverse thinking in order to cultivate creativity and produce novel solutions for intricate challenges. The CPS approach, which is rooted in the Osborn-Parnes Creative Problem-Solving (OPCPS) model, has been employed in various research endeavors and has been modified for application in educational contexts [[37], [38], [39]]. The CPS methodology offers a methodical and organized approach to addressing intricate problems, while also cultivating a mindset that is conducive to creativity and open-mindedness. The process consists of six steps: clarification, generating Ideas, analyzing ideas, developing solutions, implementing Solutions, and evaluating and reflecting.

In a study that targeted primary students with disabilities who took part in a general education classroom activities based on CPS, it was found very beneficial in increasing student communication [40]. A study by Karamustafaoğlu et al. [41] indicated that STEM activities conducted in a non-school setting enhanced students' creative problem-solving abilities and their awareness of STEM subjects. In accordance with the objectives of this research, the Creative Problem Solving (CPS) framework offered a structure for the study.

## 5 Methodology

### 5.1 Research context and participants

**Figure (1):**
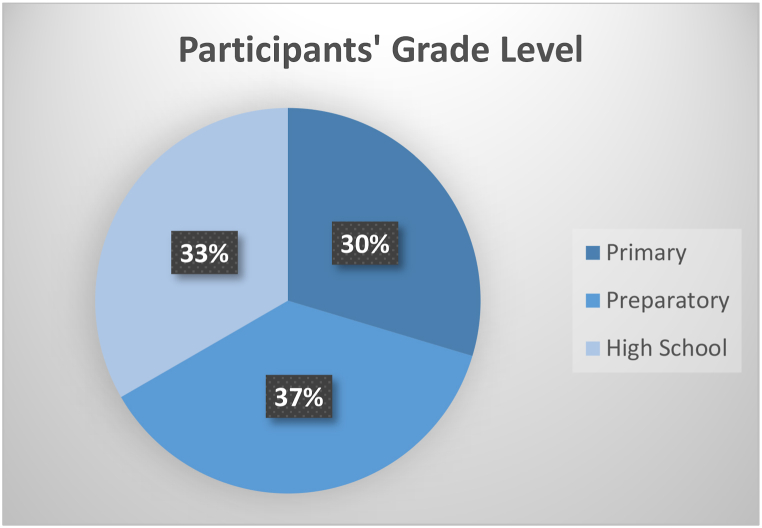
No of Deaf Students based on their grade levels.

Over five consecutive days, an interactive creative problem-solving workshop was performed for students suffering from deafness or hard of hearing. The participants in this study were selected through purposive sampling, ensuring representation from each educational level (primary, preparatory, and secondary) within the deaf student population at the participating school, as per the recommendations of schoolteachers. These recommendations are made in consideration of the participants' similar academic records, which are attributed mainly to the deafening case. In addition, it is worth noting that the workshop curriculum is in alignment with the formal school curriculum. By integrating scientific knowledge with the creative problem solving (CPS) framework, the workshop aimed to equip deaf students with problem solving skills and STEM skills, necessary to address complex challenges with innovative solutions. Participants were divided into groups of 3–4 students to reinforce daily collaboration and interaction. The workshop was designed to address a problem that causes global warming every day and would require students to apply CPS to develop innovative solutions. The hands-on activities promoted analytical thinking, deepened scientific understanding of global warming, and addressed complexities through teamwork. Through this workshop, students grasped the intricacies of global warming but also improved their ability to approach challenges systematically, emerging as capable problem solvers. At the end of the workshop, each group creates awareness posters about the problem and innovative solutions. The current study involved a cohort of 27 children with hearing impairments who were enrolled in an Audio Complex school for boys at three different educational levels ((8) at the primary level, (10) at the preparatory level, and (9) at the secondary level). The age ranges of the participants within each grade level are as follows: 12–16 years old for the primary grade level, 13–16 years old for the preparatory grade level, and 14–21 years old for the high school grade level. Figure (1) demonstrates the number of students and their distribution based on their grade level. Two mentors conducted the workshop, while four-science schoolteachers observed the implementation of the workshop. Besides, the school also provided three translators to convert the mentor's information to the sign language. The mentor's observations were recorded in problem-solving abilities and the evaluation of hands-on activities.

The effectiveness of learning models and problem solving is contingent upon the mentor's aptitude in managing learning through the utilization of a creative problem-solving (CPS) model. This was assessed using observation sheets. The efficacy of student learning can also be assessed by monitoring their activities using observation sheets. The evaluation of student engagement in environmental hands-on activities includes an assessment of their attitudes and skills.

The study was carried out in accordance with the guidelines of the Declaration of Helsinki and has received approval from the Institutional Review Board of Qatar University (QU-IRB 2108843-1). All subjects participating in the study provided informed consent.

### 5.2 Module description

The workshop was specifically designed to address the issue of global warming as a significant environmental challenge, with a particular emphasis on delivering STEM activities. The workshop syllabus comprises tasks that are designed to be comprehensible and accessible for students across all grade levels, facilitating understanding and ease of practice. According to the literature, deaf students can become aware of real-world environmental challenges by tackling the CPS framework and engaging with various workshop goals. In the current study, a STEM workshop introduced students to the phenomenon of Global Warming and its effects on our world through the presentation of real-world examples such as desertification, drought, floods, and the melting of polar ice caps. Alongside with delivering ice-breaking activities through use of images and videos to discuss the factors that leads to this phenomenon such as the accumulating of carbon dioxide in the atmosphere, which is emitted from the transportation, stationary and portable power plants, and factories. Additionally, factors such as green patch removal, waste burning, forest fires and deforestation, were presented to the students to show how these seen phenomena in our life affecting the Global Warming. The Science aspect of the topic explored the formation and accumulation of CO2 particles in the atmosphere, resulting in a rise in global temperatures. Moreover, the science behind preparing a prototype of small sample of prepared soil in which the concept of plantation should be touched. The Technology part was presented through an activity that demonstrated the process of connecting the solar cell electronic circuit. This activity aimed to enhance students' understanding of solar energy and the use of this technology if could help reducing reliance on non-renewable energy sources. The student acquired knowledge in Engineering by learning the process of designing and constructing a model house that will be integrated with a solar energy system. The Mathematical component was incorporated into the activity of exploring various transportation options, where students engage in optimizing their selection based on calculations of emissions, time savings, and comfort. Furthermore, the amount of carbon dioxide (CO2) absorbed by the planted trees were computed. Finally, the students adhere to the prescribed sequence of the CPS steps that have been previously mentioned. In each activity, students identified the problem, brainstormed of several related solutions, then selected the most appropriate option and implemented it.

### 5.3 Survey procedure

To investigate the progression of learning strategies, a post questionnaire design was employed after the workshop implementation. The post questionnaires comprised of five sections presenting the basic participants’ information in addition to the four constructs of the study. The second section was aimed to examine problem solving skills, utilizing 3-points Likert scale format [42] in which the responses were coded on a numerical scale ranging from 1 to −1. Specifically, the coding scheme assigned the value of 1 to indicate agreement with the statement, 0 to represent uncertainty or lack of knowledge, and −1 to signify disagreement. For the mentors and teachers the scale is based on 5 points (1 strongly disagree, 2 disagree, 3 neutral, 4 agree, 5 strongly agree). The total statements for teachers and mentors were five for each. The survey dimensions included aspects related to the development of STEM skills, the Evaluation of Workshop Effectiveness, and Subject Knowledge. The overall number of statements for students included (28) questions across all sections. The survey was delivered immediately after the conclusion of the workshop. The Arabic responses were subsequently translated into English in order to facilitate a seamless analysis process. Before the survey was given, the students, teachers, and people in charge of the school gave their signed permission. The survey took around 15 and 30 min to be completed.

### 5.4 Data analysis

After data was gathered, SPSS (the Statistical Package for The Social Sciences) software was used to code, save, and analyze participant responses. The data was subjected to an analysis of descriptive statistics in order to provide an overall assessment. In order to evaluate the reliability of the data we provided, various statistical tests were employed, taking into consideration the specific characteristics of the data under examination. Initially, a Cronbach Alpha and McDonald's Omega tests were conducted to assess the reliability of the questions utilized for analysis in the study. Table (1) presents the computed alpha and omega values for each survey construct. The results of the reliability test indicated that all of the questions utilized for analysis demonstrated a high level of reliability. Alpha and Omega values that exceed .70 are commonly acknowledged as indicative of reliability, while those surpassing .8 are widely regarded as indicative of a high level of reliability [43].

**Table 1:** Cronbach's Alpha and McDonald's Omega Reliability Tests for Primary, Preparatory, and High School grade levels.

Constructs	No of Items	Cronbach's Alpha	McDonald's Omega
Problem Solving skills	8	.714	.853
STEM skills	5	.752	.750
Subject Knowledge	5	.875	.893
Workshop Effectiveness	10	.830	.790

Furthermore, the normality of the data was assessed by conducting the Shapiro-Wilk and Kolmogorov-Smirnov tests, as shown in Table (2). In the context of the normality test, the dependent variable was determined to be the scores of students' of the four constructs. These scores were obtained by summing the responses of students to each of the questions within the specific construct. The independent variables in this study were the different school grade levels, specifically primary, preparatory, and high school levels. The results of the normality tests were statistically significant (p < 0.05), indicating that the distribution deviated from normality. Considering the null hypothesis positing that the data follows a normal distribution, a p-value below .05 signifies the rejection of said hypothesis. This observation provides evidence in support of our assertion that the data does not follow a normal distribution, thereby justifying the utilization of non-parametric methods for data analysis.

**Table 2:** Normality Test for the three-grade levels (Primary, Preparatory, and High School).

Dependent Variable	Independent Variable	Kolmogorov-Smirnov			Shapiro-Wilk		
		Statistic	df	Sig.	Statistic	df	Sig.
Problem Solving Skills	Primary	.301	8	.032	.774	8	.015
	Preparatory	.294	10	.014	.821	10	.026
	High School	.279	9	.042	.678	9	<.001
STEM Skills Development	Primary	.513	8	<.001	.418	8	<.001
	Preparatory	.324	10	.004	.804	10	.016
	High School	.321	9	.008	.652	9	<.001
Subject Knowledge	Primary	.455	8	<.001	.566	8	<.001
	Preparatory	.453	10	<.001	.475	10	<.001
	High School	.351	9	.002	.575	9	<.001
Workshop Effectiveness	Primary	.513	8	<.001	.418	8	<.001
	Preparatory	.205	10	.200*	.874	10	.110
	High School	.312	9	.012	.760	9	.007

Note: Df = degree of freedom; * Statistically significant; Sig. = significance level at .05.

## 6 Results

Descriptive statistics and other relevant non-parametric statistical tests were calculated to conduct a comprehensive analysis of the data, in accordance with the scope outlined in the paper [44]. To investigate the research question, the researchers computed descriptive statistics, including measures of mean, variability (standard deviation), and frequencies. A Kruskal-Wallis test was conducted to examine the scores of students' Problem-solving skills across different grade levels, including primary, preparatory, and high school. The statistical analysis revealed that the distribution of problem-solving skills is consistent Primary, Preparatory, and High School categories. The STEM skills development in primary, preparatory, and high school settings are consistent across these educational categories. This study also examines the effectiveness of workshops and their distribution across different categories of primary, preparatory, and high schools. The results are shown in Table (3). However, the mean rank for the primary and high school are 15.06 and 16.72 respectively, higher than the preparatory level in problem-solving construct. Moreover, the primary students scored a high mean rank for STEM skills development and workshop effectiveness. The distribution of Subject Knowledge is consistent across categories of Primary, Preparatory, and High School. This test concludes that all four constructs have no significant effect on the different grade levels for deaf students, which retains the null hypothesis of the research question.

**Table 3:** Non-parametric Test Analysis for Primary, Preparatory, and High School grade levels.

No	Variables	Groups	Test Statistics	Values
1	Problem Solving Skills	Primary	Mean rank	15.06
		Preparatory	Mean rank	10.70
		High School	Mean rank	16.72
			Kruskal Wallis test	3.168
			p-value	.205
2	STEM Skills	Primary	Mean rank	18.13
		Preparatory	Mean rank	10.40
		High School		14.33
			Kruskal Wallis test	5.222
			p-value	.073
3	Workshop Effectiveness	Primary	Mean rank	18.06
		Preparatory	Mean rank	11.10
		High School		13.61
			Kruskal Wallis test	4.169
			p-value	.124
4	Subject Knowledge	Primary	Mean rank	14.50
		Preparatory	Mean rank	14.75
		High School		12.72
			Kruskal Wallis test	.599
			p-value	.741

**Figure (2):**
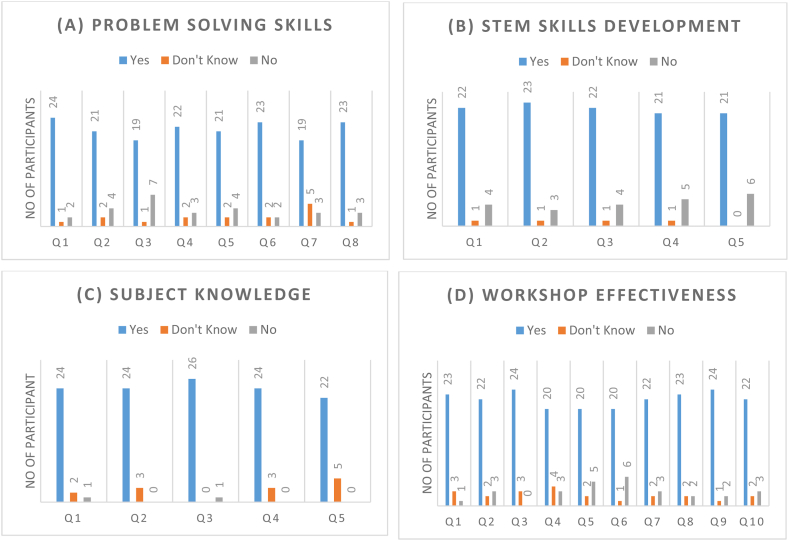
Students responses for each questions in the four constructs (A) problem-solving skills, (B) STEM skills development, (C) subject knowledge, and (D) workshop effectiveness.

Fig. 2 illustrates the participants' responses pertaining to each question within the specific construct. Concerning problem-solving skills as in figure (2-A), it is noteworthy that a majority of over 20 students have agreed with the provided statements, except for questions 3 and 7. These particular questions have garnered disagreement from many of students, indicating their reliance on teacher assistance to resolve problems in question 3, and acknowledging enhanced ease in problem-solving after attending the workshop. The questionnaire results indicate a strong agreement among students with the statements presented in the questions, suggesting a high level of engagement in the development of STEM skills as it can be seen in figure (2-B). Another notable observation is that a majority of the questions received minimal responses from students, specifically less than one student, indicating a positive trend in the development of STEM skills. The same pattern has been seen in the responses regarding the workshop's effectiveness as in figure (2-D), and the level of agreement that the students had with the statement was very high for all the questions. In the final analysis, with regard to the subject knowledge construct in figure (2-C), the responses of the students to the five questions also registered a high degree of agreement. This was especially the case for the third question, to which 99 % of the students responded that they learned new things from the workshop that they had not been familiar with previously.

**Fig. 3:**
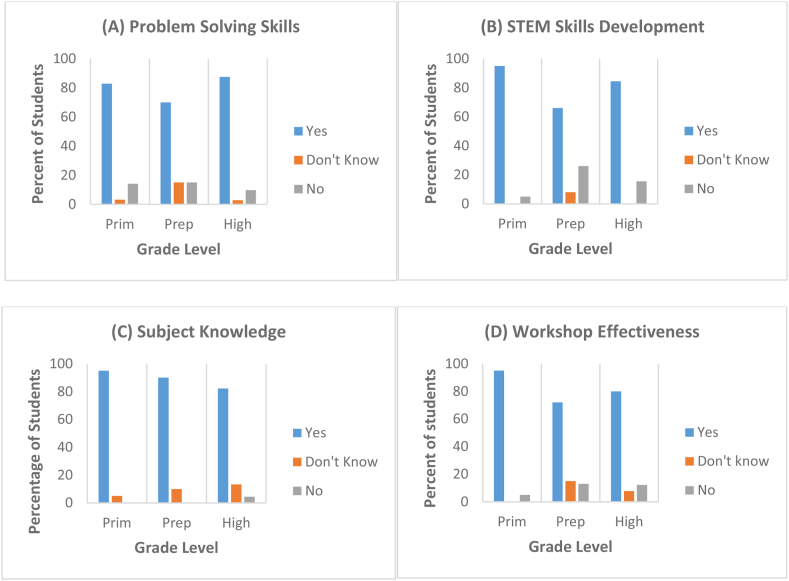
Mean scores for students perception of (A) Problem-solving Skills, (B) STEM Skills Development, (C) Subject Knowledge, and (D) Workshop Effectiveness.

Fig. 3 depicts the favorable dispositions exhibited by Deaf students across various grade levels towards the four distinct constructs. The data suggests that the primary level exhibited a higher proportion of favorable responses in relation to STEM skills (figure (3-B)), which are based on the problem solving and the effectiveness of the workshop. Conversely, in the context of preparatory grade levels, most of students responded with "Don't Know" when asked about any of the constructs (figure (3-A), (3-B), (3-C), and (3-D)). This occurrence was observed in all of the constructions. Except for their problem-solving skills figure (3-A), where their percentage was higher up to 95 %, no observations of high school students exceeding other levels have been recorded.

The bivariate correlation analysis presented in Table (4) indicates a significant relationship between the participants' Problem-solving Skills, STEM Skills Development, and Workshop Effectiveness. The Pearson correlation coefficient is .728, making it abundantly clear that an improvement in STEM skill development, which is developed based on an approach that prioritizes problem-solving significantly impacts the workshop's effectiveness. On the other hand, this does not hold true for general problem-solving abilities, for which the correlation is considered normal and the Pearson correlation coefficient is found to be .524. A slight degree of correlation has been observed between the subject knowledge construct and the other three constructs. This suggests that the workshop effectiveness will be influenced in direct proportion to the increase in awareness regarding problem-solving skills and their application in the development of STEM competencies. This phenomenon can be attributed to the strong correlation between the two variables.

**Table 4:** Bivariate correlations of the relationship between the constructs.

Construct		Problem-solving Skills	STEM Skills Development	Workshop Effectiveness	Subject Knowledge
Problem-solving Skills	Pearson Correlation	1			
STEM Skills Development	Pearson Correlation	.454^a^	1		
Workshop Effectiveness	Pearson Correlation	.524^b^	.728^b^	1	
Subject Knowledge	Pearson Correlation	−.010	−.150	−.283	1

a Correlation is significant at the .05 level (2-tailed).

b Correlation is significant at the .01 level (2-tailed).

**Fig. 4:**
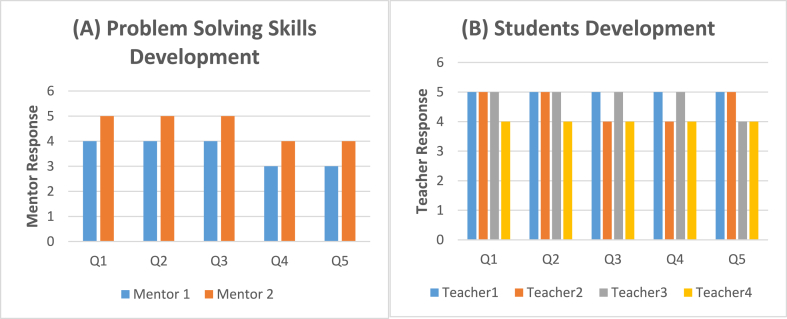
Scores for (A) mentors prospective about the students' problem-solving skills development (B) teachers prospective about students' development after the workshop.

Fig. 4 explains the mentors' responses to the questionnaire in figure (4-A), which serves as an illustration of the process by which the problem-solving abilities of students were developed. Both mentors exhibited a consistent pattern in their responses across all five questions. The data exhibited concurrence in the responses to the first three questions, while the responses to the first mentor were neutral. Conversely, the second mentor's response aligned with agreement for the fifth question. In figure (4-B), four schoolteachers present their opinions about the students' development after the workshop, their responses had followed the same trend, and their responses showed a degree of agreement to all statements.

## 7 Discussion

**Fig. 5:**
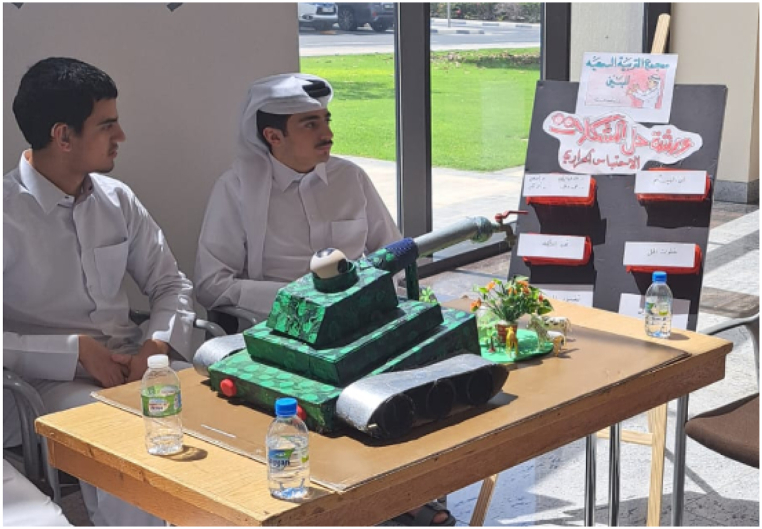
Students' creative 3D poster, which reflects deep understanding of the workshop.

Various factors, encompassing those that are unique to the experiences needs of deaf students, can exert an influence on the constructs of the STEM based workshops. These constructs include problem-solving skills, the development of STEM skills, subject knowledge, and workshop effectiveness. Comprehending these factors is of utmost importance for educators and support professionals in order to deliver efficacious assistance in science education. The findings of the data analysis indicate that there are no statistically significant differences in problem-solving constructs among deaf students of different grade levels, namely Primary, Preparatory, and High School. Additionally, the research results reveal that the problem-solving skills and STEM skill development of the students significantly influenced the workshop effectiveness. The primary outcome of the study indicates that problem-solving significantly influenced the academic performance of deaf students. This conclusion is supported by the data presented in Figure (2), Fig. 3, which demonstrate improvements across all four constructs as reported by the students. The results were consistent with the data collected during the workshop, where the mentors and teachers assessed the students’ outputs through the creation and evaluation of a creative 3D awareness poster as in Fig. 5 below, besides the assessment of the hands-on activities in the workshop. In addition, the agreement with the responses of mentors and teachers has been illustrated in Fig. 4. In general, Observing deaf students in a classroom setting requires careful attention to their unique needs and communication methods. Some factors influence the observation process for the deaf students such as familiarity with deaf culture and sign language, interpreter or communication facilitator, visual aids and technology, and collaboration with support staff [45,46].

In accordance with the observations made by the workshop mentors and schoolteachers, it was noted that the performance level of the students remained relatively consistent across all workshop participants. The educators have also verified that the academic transcripts of the students in STEM-related subjects exhibit a high degree of similarity within the designated grade level. This finding can be interpreted as indicating that there are no discernible differences between the constructs under investigation. Furthermore, it should be noted that the aforementioned school had not previously offered a workshop of this nature, specifically one that focused on developing a high-order skill such as problem-solving. This workshop provided a unique opportunity for all participants, as well as for the school itself, to engage in the practical application of STEM based techniques. However, despite the lack of significance in the non-parametric tests presented in Table (3) with regard to the differences in data between the grade levels, the mean rank provides a significant conclusion. Therefore, the findings indicate that both primary and high school students exhibited similar mean values in problem-solving skills, which were higher than the mean value observed for preparatory school students. This outcome is consistent with the graphical representations in Fig. 5, particularly in relation to the development of STEM skills. In relation to the efficacy of the workshop framework, the findings revealed a significant disparity between the responses of primary school students and those of the two other grade levels. This suggests as in Table (5) and based on the mean rank score, that it may be appropriate to introduce a STEM based workshop for primary students at the specified grade level in this particular case. Among the three grade levels, it is observed that preparatory students exhibit higher responses in the subject knowledge constructs compared to the other two grade levels. In general, from the table, the overall effectiveness of the workshop has the same effect for all three-grade levels.

**Table 5:** Effect of the four three constructs and the overall effectiveness of the workshop based on the mean rank score for the three grade levels.

	Problem-solving Skills	STEM Skills Development	Subject Knowledge	Effectiveness of Workshop
Primary	15.06	18.13	18.06	14.5
Preparatory	10.7	10.4	11.1	14.75
High School	16.72	14.33	13.61	12.72

Furthermore, the age factor is a crucial consideration in this case, in addition to the reasons previously mentioned. In regards to the age of the participants, it is important to note that a significant proportion of deaf students may commence their education at a later stage compared to their hearing counterparts. This delay can be attributed to a variety of factors [47]. It is imperative to acknowledge that the circumstances of each deaf student are distinct. The aforementioned factors may arise from a delay in identifying hearing loss, which in turn could result in a postponement of the delivery of suitable educational services. Hearing loss can be attributed to various medical conditions, including but not limited to premature birth, illness, and congenital factors. These circumstances may require specialized care and interventions prior to a child's readiness to commence formal education. Furthermore, the delay in a deaf child's school entry can be ascribed to familial circumstances, and social factors can also exert an influence on the timing of their enrollment. Variations in the timing of a child's enrollment in school may be influenced by factors such as family relocation, parental employment, or personal preferences.

In the majority of instances, the effectiveness of STEM based workshop can be significant when it is meticulously crafted and implemented. The obtained correlation results validate that, in the context of the examined case, the effectiveness is primarily influenced by two factors: most significantly, STEM skills and problem-solving skills. STEM skills are highly relevant to the workshop effectiveness in a wide range of contexts. These skills provide individuals with the tools and mindset needed to approach problems systematically, critically, and creatively. Whether in STEM-related careers or other fields, STEM skills enhance the capacity to tackle complex challenges and make informed decisions.

The research conducted by Molnar [48] revealed a significant association between workshop-based knowledge acquisition and problem-solving abilities. However, contrary to expectations, this study demonstrates that the problem-solving skills of deaf students do not have a substantial impact on their subject knowledge. The development of problem-solving skills is crucial for enhancing subject knowledge, as they offer a systematic and efficient method for acquiring and comprehending intricate concepts. The process of performing a STEM based workshop plays a central role in scientific inquiry and greatly contributes to the accumulation and progression of scientific knowledge. However, challenges arise when it comes to obtaining results and acquiring knowledge for deaf students, which can hinder their academic performance compared to their hearing peers.

The delivery of STEM workshops for deaf students can significantly affect their problem-solving skills, subject knowledge, STEM skills, and overall effectiveness based on their educational level, whether it is primary, preparatory, or secondary. This finding is supported by several studies that emphasize the importance of considering cognitive development, prior knowledge, and age-appropriate pedagogical strategies when designing and delivering STEMcontent for deaf students at different levels (Marschark et al.; Pagano et al.; Braun et al.) [7,17,21]. Marschark et al. highlight that the cognitive development and prior knowledge of deaf students at different educational levels can influence their learning outcomes in STEM education, while Pagano et al. describe a successful laboratory science technology program that tailored its content and delivery methods to the needs of deaf and hard-of-hearing students at different educational levels, leading to improved learning outcomes and increased student engagement. Additionally, research suggests that secondary-level deaf students may benefit more from STEM workshops focusing on developing problem-solving and critical thinking skills [49], while primary-level deaf students may benefit more from workshops that focus on building foundational skills and conceptual understanding through hands-on, visual, and interactive activities [50].

## 8 Conclusion

This survey-based quantitative research explored the deaf students using the creative problem-solving framework to assess different constructs such as problem-solving Skills, STEM Skills development, Subject knowledge, and general effectiveness of workshop after the implementation of a five-day STEM based workshop. The research was based on a quantitative framework. The survey results from 27 deaf students across all grade levels (primary, preparatory, and high school) revealed that all four constructs have no significant effect on the different grade levels for the deaf students, which maintains the null hypothesis of the research question. On the other hand, the primary students show a higher proportion of favorable responses in relation to problem-solving skills, STEM skills and the effectiveness of the workshop. In addition, it seemed that the proportion of neutral responses was quite high for the preparatory grade level. Finally, when it came to the ability to solve problems, the responses that were the most helpful came from high school students. It is recommended that other factors can be tested between the three grade levels such as STEM interest, gender, self-confidence 5C skills, and Family influences.

In relation to the association between the four constructs, the bivariate correlation analysis reveals a robust correlation between the effectiveness of the workshop and both problem-solving skills and the development of STEM skills. However, the analysis revealed a weak correlation between the knowledge subject and the other three constructs.

Based on the aforementioned statements, the most important conclusion is that it was imperative to explore the impact of creative problem-solving on a cohort of deaf students. The study revealed that implementing problem-solving skills, regardless of the student's grade level, could be beneficial for fostering higher-order educational abilities, such as problem-solving proficiency, STEM skill development, subject knowledge and workshop effectiveness.

Another important aspect to consider is the direct impact that enhanced problem-solving skills and STEM skills can have on the overall effectiveness of the workshop for students with hearing impairments.

The findings of this study should be interpreted with certain limitations in mind. The first is a methodological concern, namely the study's exclusive reliance on survey data. Employing a mixed-methods approach would provide a more comprehensive understanding of the topic. The workshop was limited to delivering content to all grade levels collectively, as per the school's recommendation prior to the workshop's commencement. Another constraint is that the workshop's content was designed to be suitable for participants across all grade levels, and individual participants may have engaged in different activities during the workshop. This research primarily focused on the problem-solving skills of deaf students, as well as STEM skills, subject knowledge, and the overall effectiveness of workshops for primary, preparatory, and high school grade levels. Future research could expand the scope of this study by including additional analyses to establish a comprehensive understanding of various other constructs related to problem-solving in deaf education, such as interests, gender, and family background. Furthermore, longitudinal approaches would be beneficial in understanding possible variations in students' developmental trajectories and visualizing alterations in their problem-solving abilities, STEM skills and subject knowledge over time.

## Funding

This work was not supported by any corporation.

## Data availability

Data will be made available on request.

## CRediT authorship contribution statement

**Ruba Anwar:** Writing – original draft, Methodology, Data curation. **Abubaker M. Elbashir:** Writing – review & editing, Writing – original draft, Visualization, Validation, Methodology, Investigation, Funding acquisition, Formal analysis, Data curation, Conceptualization. **Rana Magdy:** Data curation, Conceptualization. **Zubair Ahmad:** Visualization, Supervision, Investigation, Formal analysis, Data curation, Conceptualization. **Noora J. Al-Thani:** Supervision, Project administration, Funding acquisition.

## Declaration of competing interest

The authors declare that they have no known competing financial interests or personal relationships that could have appeared to influence the work reported in this paper.
