# Supplementary material for: Effectiveness of STEM based workshop for deaf education: Exploratory study
Source: Heliyon. 2024 Aug 10;10(16):e36012. doi: 10.1016/j.heliyon.2024.e36012 (PMC11357752; doi:10.1016/j.heliyon.2024.e36012)

1. **Students’ Questionnaire**

**Problem Solving Program**

**Module Name: *Global Warming***

**Thank you for filling this survey. Kindly read the statements carefully before you response**

| **PART I: STUDENT’S DEMOGRAPHIC PROFILE** |
| --- |

1. **Name …………………………………………………………….**
2. **Gender**
3. Male
4. Female

1. **Date of Birth**

(Day/ Month/ Year) **____ / ____ / ____**

1. **Nationality**
2. Qatari
3. Non-Qatari

1. **School Type**
2. Government school / Independent school
3. International / Private school
4. **School Name: .............................................................................**
5. **Grade: .............................................................................**

| **PART II: PROBLEM-SOLVING SKILLS** |
| --- |

**Q8. Please read the following statements and answer choose the most correct answer.**

(Yes, No, Don’t Know)

| **Statement** | **Yes** | **No** | **Don’t know** |
| --- | --- | --- | --- |
| 1. I can easily identify scientific problems |  |  |  |
| 1. I usually need my teacher’s assistance to identify scientific problems |  |  |  |
| 1. I face obstacles in understanding scientific problems |  |  |  |
| 1. I spend too much time understanding scientific problems |  |  |  |
| 1. I easily find out the solution to scientific problems |  |  |  |
| 1. I usually need my teacher’s assistance to solve scientific problems |  |  |  |
| 1. I know all the problem-solving steps to solve a scientific problem |  |  |  |
| 1. I am confident that I can solve scientific problems |  |  |  |

| **PART III: STEM SKILLS DEVELOPMENT** |
| --- |

**Q9. Please read the following statements and answer choose the most correct answer.**

(Yes, No, Don’t Know)

| **Statement** | **Yes** | **No** | **Don’t know** |
| --- | --- | --- | --- |
| 1. Problem-solving workshop helped me to know the step to solve a scientific problem |  |  |  |
| 1. Problem-solving workshop helped me to understand maths |  |  |  |
| 1. Problem-solving workshop helped me to understand technology |  |  |  |
| 1. Problem-solving workshop helped me to understand engineering |  |  |  |
| 1. Problem-solving workshop helped me to improve my problem-solving skills |  |  |  |

| **PART IV: EFFECTIVENESS OF WORKSHOP** |
| --- |

**Q9. Please read the following statements and answer choose the most correct answer.**

(Yes, No, Don’t Know)

| **Statement** | **Yes** | **No** | **Don’t know** |
| --- | --- | --- | --- |
| 1. Global Warming workshop helps me to understand scientific problems |  |  |  |
| 1. I solved the scientific problems using a problem-solving workshop |  |  |  |
| 1. Learning through a problem-solving workshop makes it easier to understand the scientific problem |  |  |  |
| 1. I am comfortable learning using the Problem-solving workshop |  |  |  |
| 1. The Problem-solving workshop helped me complete the tasks and activities effectively |  |  |  |
| 1. My experience of the Problem-solving workshop was better than I expected |  |  |  |
| 1. I would like to participate in more Problem-solving workshops |  |  |  |
| 1. Using Problem-solving workshop is a good to add to the conventional teaching methods |  |  |  |
| 1. I would like to share my Problem-solving workshop experience with others |  |  |  |
| 1. I am excited to attend the next cycle of the Problem-solving workshop |  |  |  |

| **PART V: SUBJECT KNOWLEDGE** |
| --- |

**Q10. Please read the following statements and answer choose the most correct answer.**

(Yes, No, Don’t Know)

| **Statement** | **Yes** | **No** | **Don’t know** |
| --- | --- | --- | --- |
| 1. Problem-solving workshop increased my knowledge on the Global Warming |  |  |  |
| 1. Problem-solving workshop helped me understand complex concepts about the Global Warming |  |  |  |
| 1. I learned new things using the Problem-solving workshop, which I did not know before about the Global Warming |  |  |  |
| 1. Problem-solving workshop removed my doubts about the Global Warming |  |  |  |
| 1. I feel confident in answering questions about the Global Warming which I learned |  |  |  |

1. **Teachers Observations**

Please state your agreement on the following statements that demonstrate development in students’ attitudes/learning behavior.

| Sr.No | Statement | Strongly Agree | Agree | Neutral | Disagree | Strongly Disagree |
| --- | --- | --- | --- | --- | --- | --- |
|  | Problem-solving workshop helps the students in developing their knowledge on the integration of science, mathematics, technology, and engineering |  |  |  |  |  |
|  | Problem-solving workshop has increased students’ knowledge on the topic (Environmental awareness, robotics, electronics etc) |  |  |  |  |  |
|  | Students learned new things using the Problem-solving workshop, which they did not know before about the topic |  |  |  |  |  |
|  | Students have developed an interest in inventing useful things |  |  |  |  |  |
|  | PS workshop aided students in developing skills to solve the scientific problem effectively |  |  |  |  |  |

1. **Mentor Observations**

| **Facilitator observation based response (Strongly agree, Agree, Neutral, Disagree, Strongly disagree)** | | | | | | | |
| --- | --- | --- | --- | --- | --- | --- | --- |
| Sl.No | Competences | Indicators | Strongly agree | Agree | Neutral | Disagree | Strongly Disagree |
| 1 | Skill Development | The students complete the assignments regularly |  |  |  |  |  |
| 2 |  | I share extra topic-related content (i.e., extra links, readings etc.) to enhance their knowledge |  |  |  |  |  |
| 3 |  | The students regularly ask clarification on scientific content that is off the lesson plan |  |  |  |  |  |
| 4 |  | The students regularly asks for extra learning materials to assist in their assignments |  |  |  |  |  |
| 5 |  | The students complete works/ assignments on time. |  |  |  |  |  |

1. **STEM activities in the workshop**

a- Activity One

What the difference between the images below:


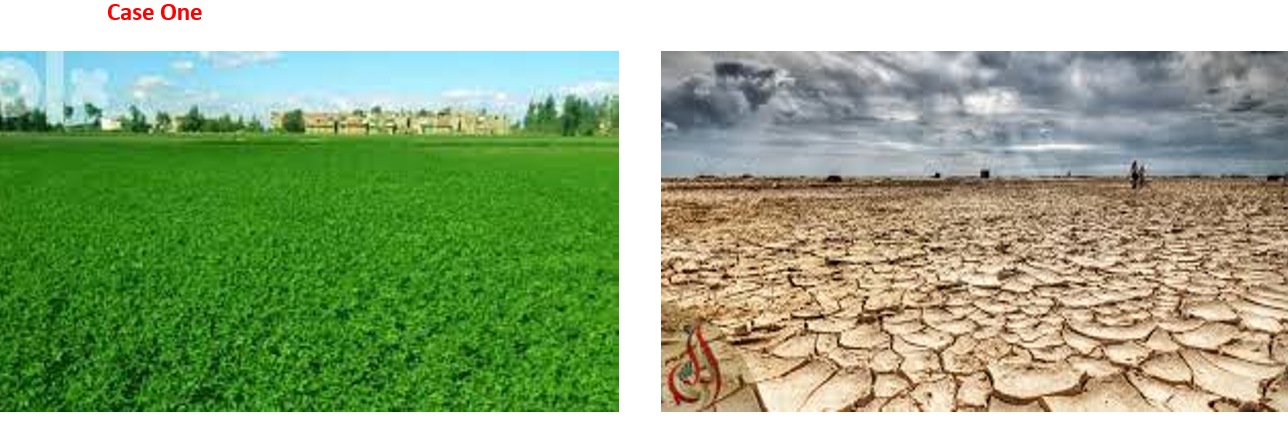


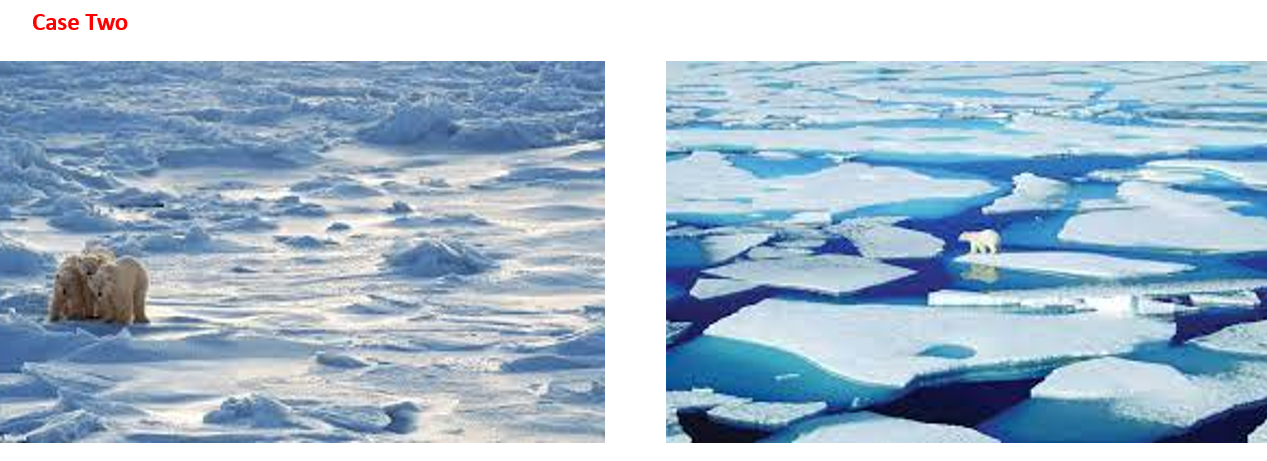


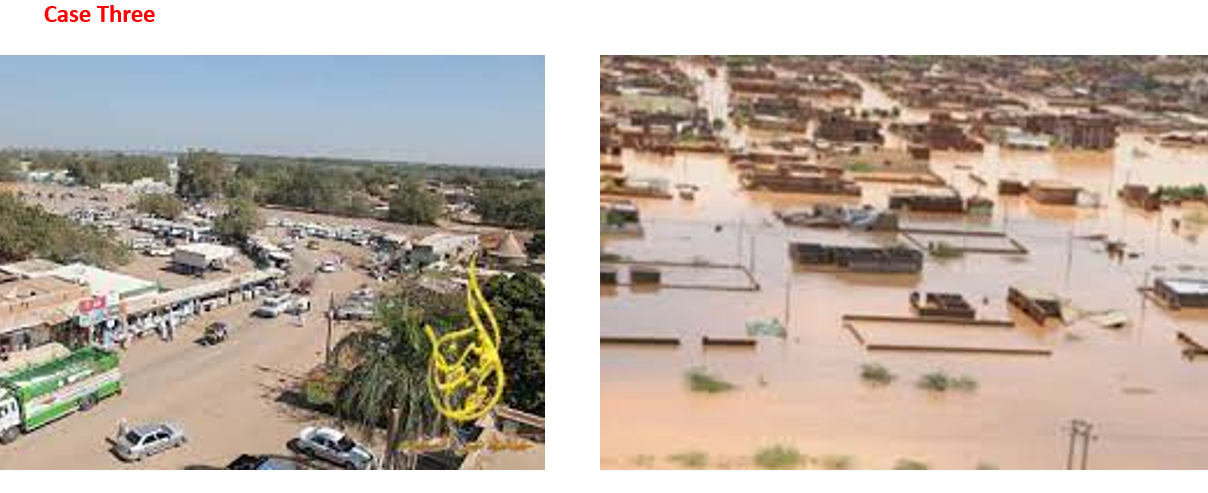


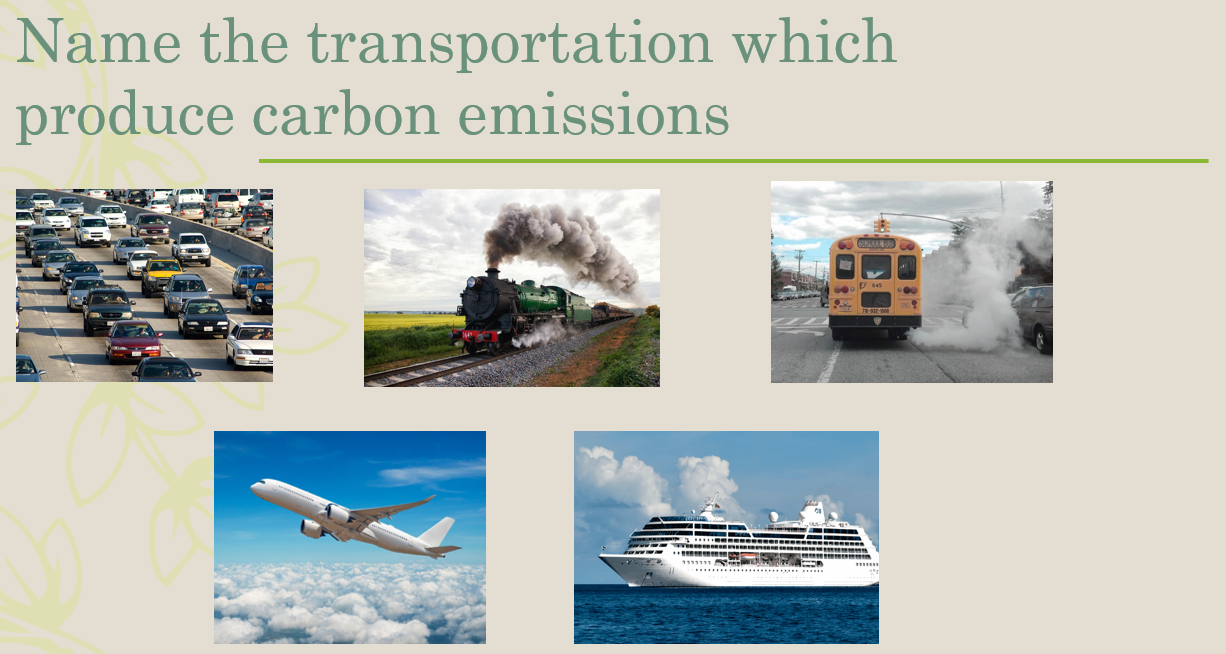


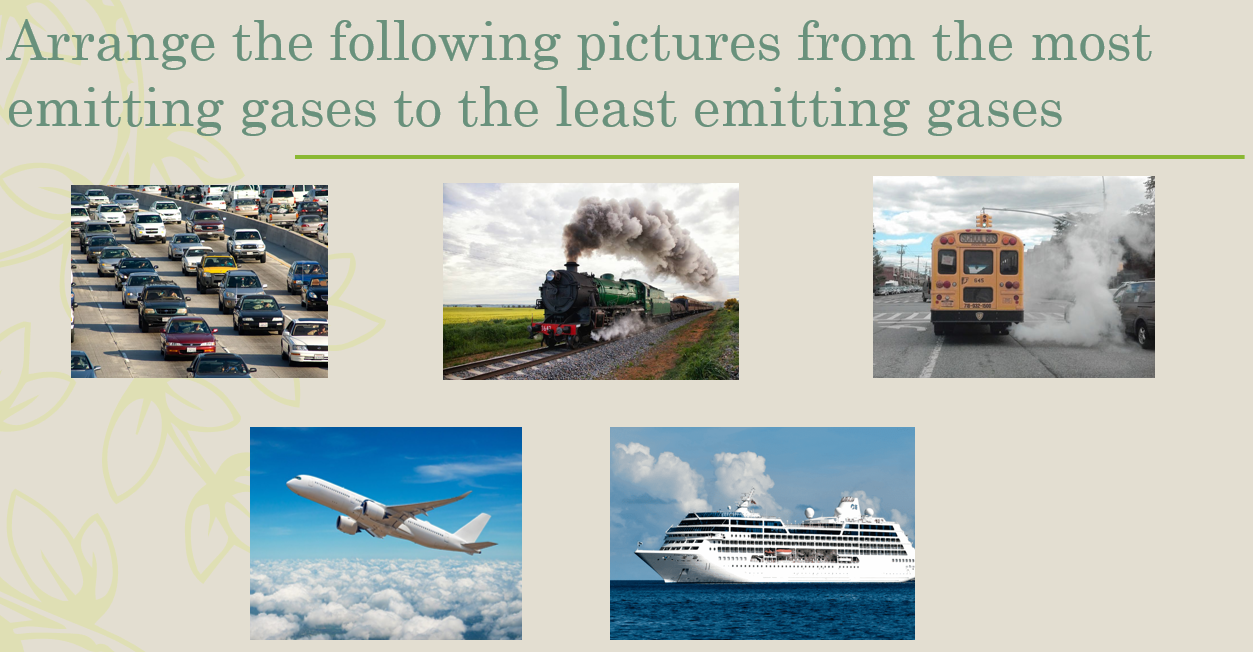


b- Activity Two


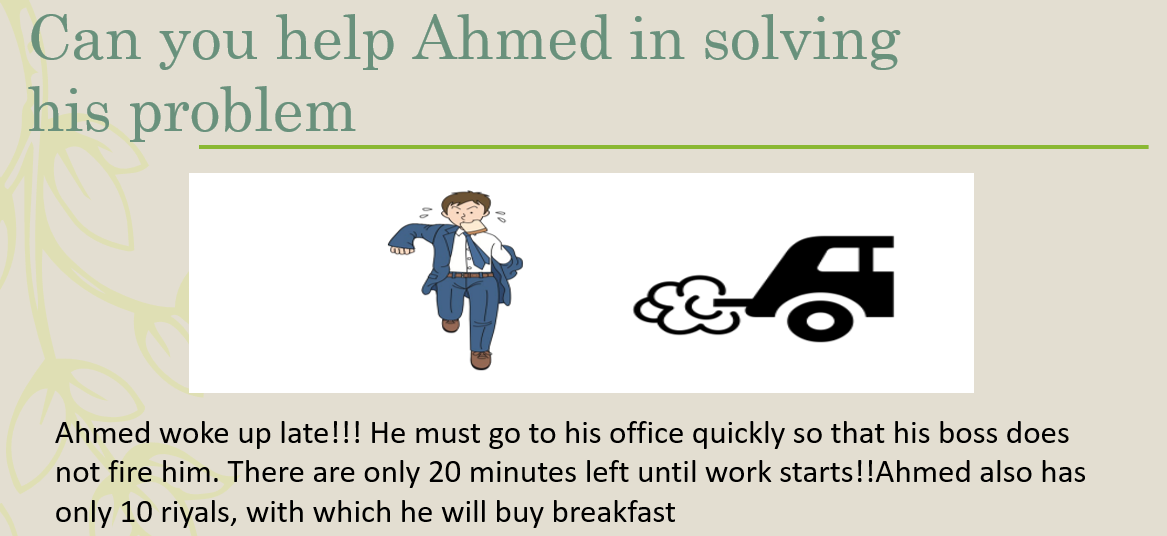

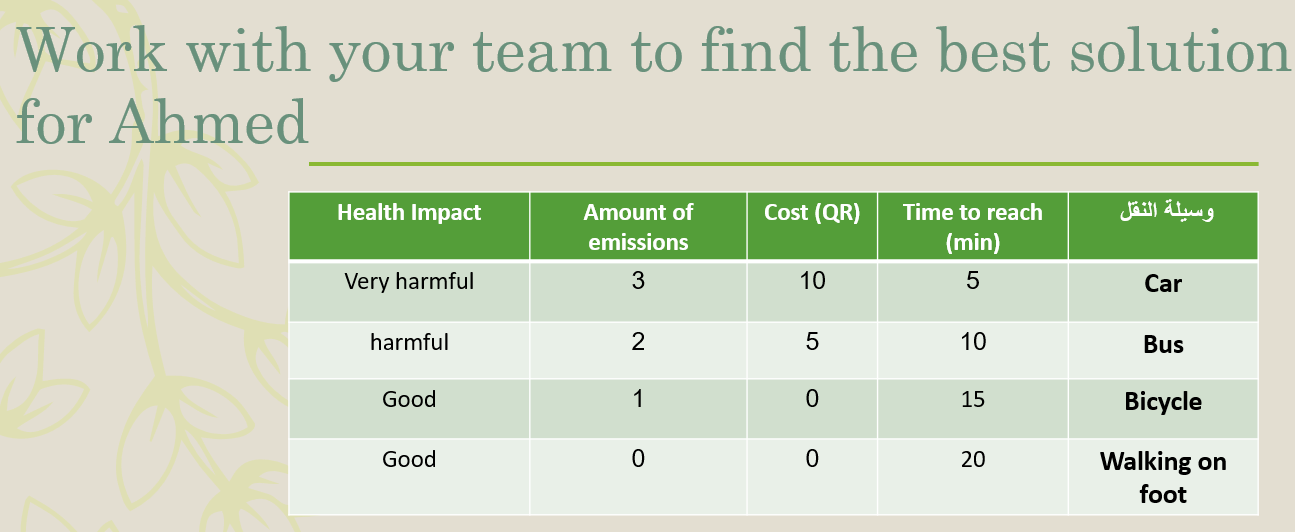


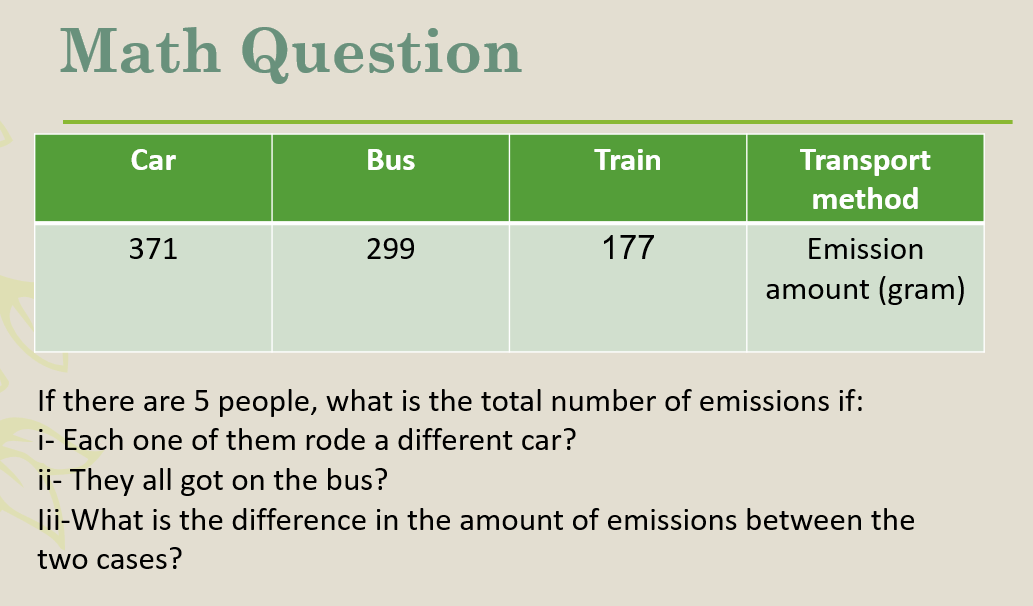


c- Activity Three (Use food waste in agriculture)


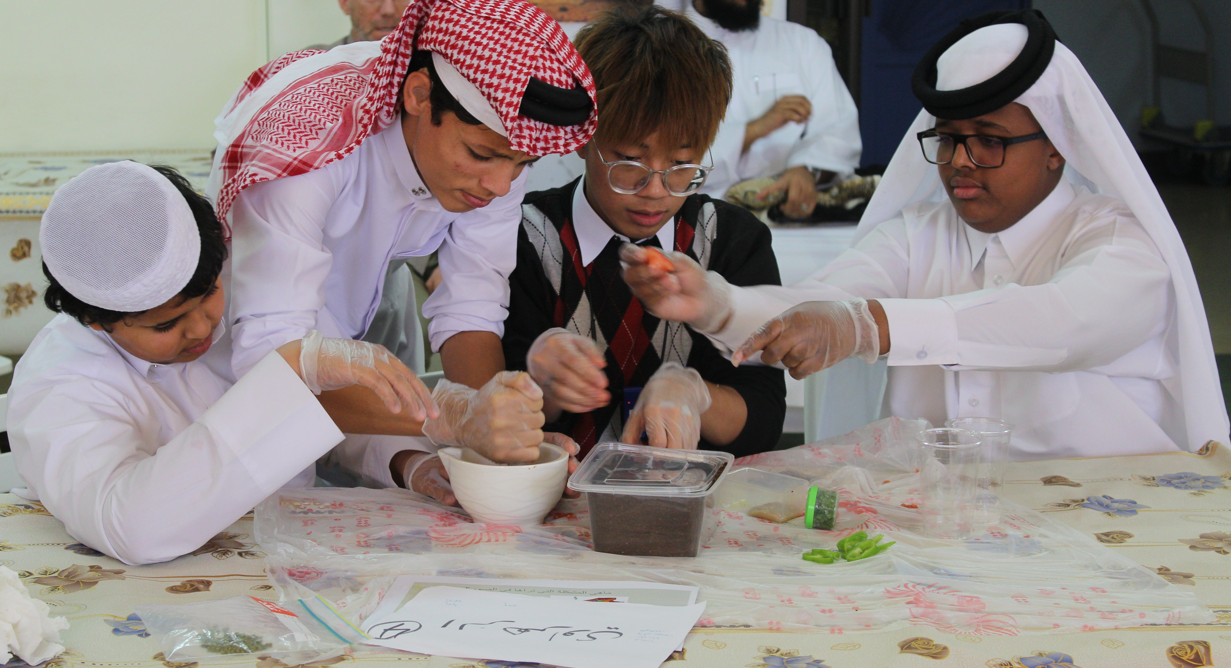


d- Activity Four (Connecting the Solar PV circuit)


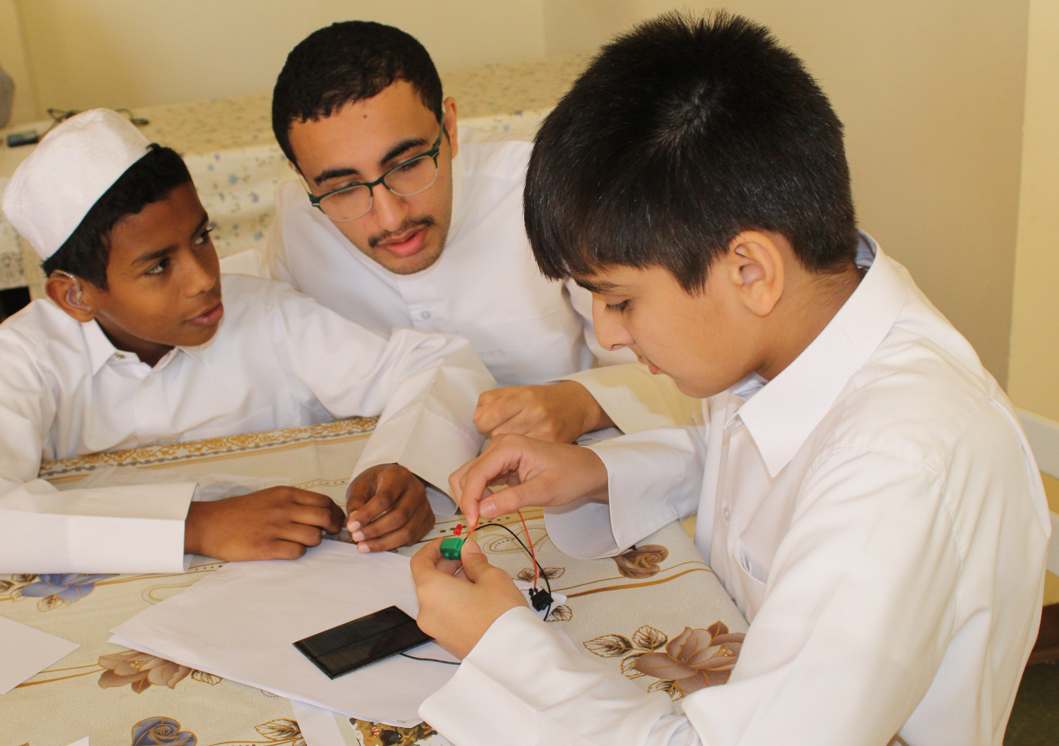

Supplement: Multimedia component 1 [file mmc1.docx]
